# Cognitive deficits in older adults with mild cognitive impairment in a two-year follow-up study

**DOI:** 10.1590/1980-57642018dn12-010003

**Published:** 2018

**Authors:** Camila de Assis Faria, Heloisa Veiga Dias Alves, Eduarda Naidel Barboza e Barbosa, Helenice Charchat-Fichman

**Affiliations:** 1 MD. Pontifícia Universidade Católica do Rio de Janeiro, RJ, Brazil; 2 PhD. Pontifícia Universidade Católica do Rio de Janeiro, RJ, Brazil

**Keywords:** mild cognitive impairment, dementia, cognitive decline, conversion, cognitive trajectory, comprometimento cognitivo leve, demência, declínio cognitivo, conversão, trajetória cognitiva

## Abstract

Characterizing cognitive decline in older adults with MCI over time is important to identify the cognitive profile of those who convert to dementia.

**Objective:**

This study examined the two-year cognitive trajectory of elderly adults diagnosed with MCI, from geriatrics and neurology outpatient clinics of a public hospital in Rio de Janeiro.

**Methods:**

62 older adults with MCI were submitted to a neuropsychological battery and re-evaluated after two years. The Mann-Whitney U test was employed to assess differences between groups with respect to education, functioning, the Geriatric Depression Scale and diagnosis.

**Results:**

24.2% converted to dementia after two years. The group with declines in two or more cognitive functions had a higher conversion rate to dementia than the group with decline in executive functions (EF) only (Z = -2.11, p = .04). The EF decline group had higher scores on the depression scale than both the memory decline group (Z = -1.99, p = .05) and multiple decline group (Z = -2.23, p = .03).

**Conclusion:**

The present study found different cognitive decline profiles in elderly adults with MCI and differences between them regarding depressive symptoms and rate of conversion to dementia.

## RESUMO

Caracterizar o declínio cognitivo em idosos com Comprometimento Cognitivo Leve (CCL) ao longo do tempo é importante para identificar o perfil cognitivo de quem converte para demência.

**Objetivo:**

Estudar as trajetórias evolutivas de dois anos de acompanhamento de idosos com CCL assistidos em clinicas de geriatria e neurologia de um Hospital Federal na cidade do Rio de Janeiro.

**Métodos:**

62 idosos com CCL foram submetidos a uma bateria neuropsicológica e reavaliados após dois anos. O teste U de Mann-Whitney foi utilizado para avaliar as diferenças entre os grupos quanto a escolaridade, funcionalidade, escala de depressão geriátrica e diagnóstico.

**Resultados:**

24,2% converteram-se em demência após dois anos. O grupo com declínio em duas ou mais funções cognitivas apresentou maior porcentagem de conversão para demência do que o grupo com declínio apenas nas funções executivas (Z = -2,11, p = 0,04). O grupo de declínio das FEs, apresentou escores maiores na escala de depressão do que o grupo de declínio na memória (Z = -1.99, p = .05) e múltiplos declínios (Z = -2.23, p = .03).

**Conclusão:**

O presente estudo mostrou que existem perfis diferentes de declínio cognitivo em idosos com CCL e que existem diferenças entre eles em relação a sintomas depressivos e a taxa de conversão para demência.

Mild cognitive impairment (MCI) is characterized by deficits in cognitive function and a related loss in the ability to perform advanced activities of daily living, such as working.^1^^,^ 2 MCI can be classified as amnestic (when there is memory impairment), amnestic multiple domains (when there is memory loss and loss in other cognitive functions), non-amnestic (when there is loss of a cognitive function other than memory), and non-amnestic multiple domains (when there is loss of other cognitive functions other than memory).^1^^,^ 2 MCI can be a transition stage to dementia, such as Alzheimer's disease or vascular dementia; however, elderly adults with MCI may improve or remain stable over the course of years.^2^

According to the DSM-IV,^3^ the diagnostic criteria for dementia include memory changes (learning and learned content recall) and aphasia or apraxia, agnosia, and changes in executive functions affecting social or work activity. Petersen et al.^4^ reported that in memory research centers and treatment clinics, the percentage of MCI conversion to dementia is approximately 10%-15% a year. Farias et al.^5^ analyzed 111 elderly adults with MCI in California (46% from a clinical population and 54% from the community) and observed an annual conversion rate of 13% in older adults treated at memory clinics and 3% in community-dwelling older adults. Epidemiological studies have reported lower annual conversion rates, from 6% to 10%, probably due to the cognitive heterogeneity found at baseline in these studies.^4^

Individuals with MCI make up a group with a high probability of conversion to dementia, when compared to healthy older adults.^4^ Cloutier et al.^6^ evaluated the trajectory of decline in cognitive functions and compared individuals who converted to dementia with older adults who did not. The delayed recall of episodic memory, working memory and spatial memory had a stable profile with a sharp decline before conversion to dementia. Immediate recall, executive functions and visuoconstructive skills had a profile of gradual decline before conversion to dementia and language had a stable profile.^6^ There are few longitudinal studies on the trajectory of cognitive decline in older adults with MCI.^6^^,^ 7 Studying cognitive decline in elderly adults with MCI over time is important to identify the cognitive profile of those who convert to dementia. This study examined the two-year cognitive trajectory of elderly adults diagnosed with MCI, from geriatrics and neurology outpatient clinics of a public hospital in Rio de Janeiro.

## METHODS

### Baseline sample characteristics

Sixty-two elderly adults with MCI treated at geriatrics and neurology outpatient clinics of a public hospital in Rio de Janeiro were submitted to a broad neuropsychological battery. All participants were diagnosed with MCI by medical experts at baseline, with no subtype specification, according to the following diagnostic criteria: subjective complaint of memory decline, confirmed by an informant; cognitive deficits indicated by tests (performance on the Mini-Mental State Examination^8^ (MMSE), Verbal Fluency Test - Animals Category,^9^ and Mini-Cog);^10^ preserved global cognitive functioning; intact functional activities (social-occupational); and absence of dementia.^11^ For MCI diagnosis, imaging tests were not ordered by doctors. However, laboratory tests were used to rule out other diseases that could cause cognitive decline. When dementia or vascular changes were suspected, imaging tests were performed.

Since the sample was selected in geriatrics and neurology outpatient clinics, it included individuals with cardiovascular disease, hypertension and/or controlled diabetes; corrected sensory deficits; and symptoms of depression or previous depressive episodes, with current mood controlled by medication and/or psychotherapy. Patients that met DSM-IV^3^ criteria for dementia; had current or previous presence of neurological diseases; severe psychiatric disorders; severe systemic disease that could lead to cognitive decline; recent history of alcoholism or drug addiction; and uncorrected visual and/or hearing deficits were excluded.

### Follow-up procedures

After two years, the older adults were contacted by telephone and reassessed. Data were collected from the medical records of each individual and the updated diagnosis established by medical experts was checked. Participants were diagnosed as having dementia according to DSM-IV^3^ criteria; imaging and laboratory results confirmed the diagnosis.

### Ethical issues

The study was submitted to the hospital's local Ethics Committee (CEP/HFSE) and was approved on May 12th, 2008 (protocol 000.320). All participants received an oral and written explanation about the purpose of the research, willingly agreed to participate, and signed an Informed Consent form prior to the beginning of the study.

### Instruments

The instruments described below were employed at baseline and at follow-up (two years later).

*Functional Activities Questionnaire (Pfeffer):* 12 Evaluates the performance of older adults in activities of daily living based on 10 questions. Each question is attributed a score ranging from 0 to 3. Total score on the scale ranges from 0 to 30. If the individual attains five points or more, he/she is considered as having an impairment in instrumental activities of daily living. Individuals with a score of 5 or more were not excluded.

*Memory for Figures:* 10 simple figures are shown to the participant and the following tasks are performed: Naming, Incidental Memory, Immediate Memory 1 and 2, Late Recall after five minutes (M5) and Late Recall with clue (Recognition).

*Clock Drawing Test (CDT):* 13 The test consists of drawing the face of a clock on a blank sheet. Total score ranges from 1 to 10 points.

*Coding Task (WAIS-R):* 14 Assesses working memory and processing speed, evaluating correlations between numbers and symbols in a 2-minute period.

*Mattis Dementia Rating Scale:* 15 ^,^ 16 Consists of tests that evaluate cognitive functioning. It contains the following subtests: Attention, Initiation/Perseveration, Construction, Conceptualization, Memory, Total Score (sum of subtests).

*Verbal Fluency Test (VFT) - phonemic FAS:* 9 The test consists of producing as many words as possible beginning with F, A and S in 1 minute. Participants cannot use proper nouns or a stem word with different endings.

*Geriatric Depression Scale (GDS-30):* 17 Assesses the degree of depressive symptoms, from mild to severe. Thirty questions (yes or no answers) assess how the subject felt in the two weeks prior to the test day. The cut-off point^17^ is 14. Individuals with scores of 14 or more are considered as having a high probability of exhibiting symptoms of depression. However, these individuals were not excluded from the study.

*Digits Forward and Backward (WMS-R):* 9 ^,^ 18 ^,^ 19 Digits Forward subtest: the participant must repeat the numbers spoken by the examiner in the same order. Digits Backward subtest: the participant must repeat the same numbers in reverse order. The scores on the two parts of this test are obtained separately and the sum of the two subtests yields the total score.

*Extension (Span) of Visuospatial Digits Forward and Backward (WMS-R):* 9 ^,^ 18 ^,^ 19 The Forward subtest consists of repeating a sequence of visuospatial items in the same order. The Backward subtest consists of repeating a sequence of visuospatial items in reverse order. The scores on the two parts are obtained separately and the sum of the two subtests yields the total score.

*Rey Auditory Verbal Learning Test (RAVLT):* 20 Consists of a list of 15 words that is repeated five times. It includes the following tasks: Repeat list five times (A1 to A5), total learning, recall after 30 minutes (A7), recall with clue (recognition).

### Statistical analysis

All analyses were performed using the SPSS software (version 18.0) for Windows. The difference between the standardized z-scores for each neuropsychological test at baseline and follow-up (re-evaluation) was calculated. Differences of 1 standard deviation were considered as indicative of decline. Descriptive analyses were conducted for the different profiles of cognitive decline observed. The Wilcoxon test was used to identify changes in functioning, on the GDS and neuropsychological testing after two years. The Mann-Whitney U test was used to identify differences between groups with respect to gender, age, education, functioning, GDS and diagnosis after two years.

## RESULTS

Differences were found between baseline and two-year follow-up on neuropsychological tests, functioning and depression symptoms, for participants who converted to dementia and those who did not.

Of the 62 elderly adults in the sample, 15 (24.2%) converted to dementia after two years according to the medical specialists. Older adults who did not convert to dementia had lower scores on time and space orientation, simple figure naming and categorical verbal fluency (clothes category). This group showed improvement in recall when given clues in the visual episodic memory measure. Older adults who converted to dementia had lower scores on recall with clues in visual episodic memory and categorical verbal fluency (supermarket category). There were no differences in functioning and depression symptoms. The Wilcoxon test results, with means at baseline and follow-up, are shown in Table 1.

**Table 1:** Wilcoxon test results: mean scores at baseline and two-year follow-up of participants converting and not converting to dementia.

		Z (p)	Mean (SD)	
			Baseline	2-year follow-up
**Not converted (N = 47)**	Time Orientation	2.17 (0.03*)	3.57 (0.75)	3.49 (0.88)
	Space Orientation	6.56 (0.00**)	1.98 (0.15)	1.94 (0.32)
	Naming	5.54 (0.00**)	9.91 (0.45)	9.89 (0.47)
	Recall with clue visual episodic memory	3.26 (0.00**)	9.17 (0.76)	9.36 (1.71)
	Clothes	3.35 (0.00**)	7.80 (0.66)	7.77 (0.69)
**Converted (N = 15)**	Recall with clue visual memory clue	1.94 (0.05*)	8.33 (2.58)	7.20 (2.88)
	Supermarket	2.35 (0.02*)	15.00 (4.72)	10.93 (4.51)

* p≤ .05;

** p≤ .001

### Characterization of neuropsychological decline after two years

The difference between the standardized scores (z), at baseline and after two years, for each neuropsychological test was calculated for the entire sample. As described in the Methods section, a decrease of 1 SD on the neuropsychological tests was considered a decline. Four different decline profiles were identified: 4 participants (6.5%) showed no decline on any of the neuropsychological tests (No Decline Group); 9 participants (14.5%) showed decline only on episodic memory or working memory storage (Memory Decline Group). Ten participants (16.1%) showed decline in executive functions only (Executive Functions Decline Group). Most participants (39 -62.9%) showed decline in two or more cognitive functions (Multiple Functions Decline Group). Table 2 illustrates the characteristics of each decline profile group.

**Table 2:** Cognitive functions that declined in each profile group (N = 62).

	Recall with clue	Learning	Recall without clue	WM Storage	Space Orientation	Time Orientation	WM Processing	Verbal Fluency	Visuospatial Planning	Attention	Language	Global cognitive functioning
	**No Decline Group**											
1												
2												
3												
4												
	**Memory Decline Group**											
5	x											
6	x											
7			x									
8		x		x								
9*				x								
10*			x	x	x	x						
11				x								
12				x								
13	x		x									
	**Executive Functions Decline Group**											
14							x					
15							x					
16								x				
17								x				
18							x		x			
19							x	x				
20							x	x	x			
21								x				
22								x				
23								x	x			
	**Multiple Functions Decline Group**											
24	x			x			x	x		x		x
25*		x	x	x			x	x	x		x	x
26*		x	x	x		x	x	x	x	x	x	x
27*	x			x	x	x	x		x		x	x
28	x		x	x				x				
29*	x		x	x			x	x	x			
30*	x						x		x			
31	x	x		x			x	x	x	x		
32	x			x							x	
33	x	x		x		x		x				
34	x			x					x			
35*				x		x					x	
36				x				x				
37			x	x				x				
38*		x					x					
39				x					x			
40		x	x				x					
41*		x		x					x			
42			x	x				x				
43			x	x				x				
44			x	x				x				
45				x			x	x				
46		x		x				x			x	
47			x	x					x	x		
48				x				x	x			
49*		x		x	x		x	x				
50*		x						x			x	
51*			x	x				x	x			
52		x		x					x		x	
53			x	x				x	x			
54		x					x	x				
55*		x	x	x			x	x	x			
56		x	x	x			x	x	x			
57				x			x	x	x		x	
58				x			x	x	x	x		
59							x				x	
60							x	x	X		X	
61				x							X	
62*							x	x		x		

* individuals who converted to dementia; WM, working memory.

### Functioning and depression symptoms across the four cognitive decline profiles at follow-up

The Wilcoxon test revealed that Group 3 (Z = 2.00, p = 0.05) had significantly lower functional scores after two years. The other results did not reach statistical significance. The means, standard deviations and medians of functioning and depression symptoms at baseline and follow-up for the four cognitive decline profiles are presented in Table 3.

**Table 3:** Rate of conversion to dementia and sociodemographic, functioning and depression symptom characteristics of the four cognitive decline groups.

	No decline group (%)	Memory decline group (%)	Executive functions decline group (%)	Multiple functions decline group (%)
Converted to dementia	0.0	22.2	0.0	33.3*
Female	100.0	66.7	70.0	71.8
	**Mean (SD)**	**Mean (SD)**	**Mean (SD)**	**Mean (SD)**
Age	78.00 (6.27)	76.44 (8.81)	77.20 (5.67)	76.74 (6.81)
Education	8.50 (6.75)	5.33 (5.24)	6.10 (2.60)	5.31 (4.04)
ADLs baseline	3.25 (2.75)	4.13 (2.99)	5.67 (5.70)	5.23 (5.53)
ADLs after 2 years	3.75 (3.30)	4.00 (3.27)	4.13 (2.94)	6.73 (6.69)
ADLs difference	0.01 (0.16)	-0.09 (0.63)	-0.48 (1.11)	0.15 (0.79)
GDS baseline	14.00 (4.83)	8.38 (6.11)	12.80 (5.80)	10.08 (6.03)
GDS after 2 years	11.00 (8.36)	8.56 (6.36)	14.89 (6.07)*	9.48 (5.68)
GDS difference	-0.46 (1.25)	0.01 (0.26)	0.42 (0.85)	0.01 (0.75)

* Significant difference between the groups (data in text); ADLs, Activities of daily living; GDS, geriatric depression scale.

### Description of the four cognitive decline profiles

*No Decline Group:* This group had no decline in the neuropsychological tests. At baseline, 50% of the subjects in this group had a deficit (SD ≤ 1 on standardized scores) in episodic memory and other cognitive functions, 25% had a deficit in episodic memory only and 25% exhibited no deficits. None of the subjects showed baseline deficits in functioning or depression symptoms. After two years, 25% showed a worsening of depressive symptoms and none converted to dementia.

*Memory Decline Group:* Older adults in this group showed a decline in episodic and short term memory storage. At baseline, 44.4% of subjects had deficits in episodic memory and other cognitive functions, 33.3% had deficits in executive functions and other cognitive functions (except memory), and 22.2% had no deficits. At baseline, 11.1% had depressive symptoms. After two years, 11.1% showed a worsening of functioning and the rate of conversion to dementia was 22.2%. The subjects who converted to dementia had baseline deficits in episodic memory and other cognitive functions (global cognitive functioning, working memory storage, executive functions, language, and attention).

*Executive Functions Decline Group:* Older adults in this group showed a decline in executive functions only. At baseline, 50% of subjects had deficits in episodic memory and other cognitive functions (especially executive functions), 20% had deficits in executive functions and other cognitive functions (except memory), and 30% showed no deficits. After two years, 20% showed a worsening in functioning and 10% in depression symptoms. None converted to dementia.

*Multiple Functions Decline Group:* This group showed a decline in two or more cognitive functions. At baseline, 51.3% of subjects had deficits in episodic memory and other cognitive functions, 5.1% a deficit in episodic memory only, 23.1% had deficits in executive functions and other cognitive functions (except memory), 7.7% had a deficit in other different cognitive functions of memory and executive functions, and 12.8% had no deficits. At baseline, 25.6% had depressive symptoms. After two years, 2.6% showed a worsening of functioning and 5.1% of depression symptoms. The rate of conversion to dementia was 33.3%. Of the 13 subjects in this group who converted to dementia, 84.6% had baseline deficits in episodic memory and other cognitive functions (global cognitive functioning, working memory storage, executive functions, language, and attention), 7.7% had deficits in executive functions and other cognitive functions (except memory), and 7.7% had a deficit in time and space orientation.

### Differences between the four cognitive decline profiles: sociodemographic, functioning and depression symptoms

The Mann-Whitney test revealed that the group with multiple functions decline showed a higher conversion rate to dementia than the group with executive function decline only (U = 130.0, p = 0.04). For median score on the GDS, the group showing a decline in executive functions only had a higher score than the group with memory decline only (U = 18.0, p = 0.05; data after 2 years) and the group with decline in two functions or more (U = 71.0, p = 0.03; data after 2 years). There were no differences for gender, age, education, and functioning across the four cognitive decline profiles. The mean, standard deviation and median values for age, educational level, functioning, and depressive symptoms of the four groups are shown in Table 3.

## DISCUSSION

This study examined a two-year cognitive trajectory of elderly adults diagnosed with MCI, from geriatrics and neurology outpatient clinics of a public hospital in Rio de Janeiro. During the follow-up period, 24.2% had converted to Alzheimer's disease. This is consistent with findings in clinical populations.^4^^,^ 5

Initially, we analyzed the difference between baseline and follow-up (two years later) for neuropsychological measures, functioning, and depression symptoms in elderly adults who converted to dementia and those who did not, according to the diagnosis of medical specialists. After two years, both groups showed significant worsening of executive functions (verbal fluency), according to the Wilcoxon test. Those who converted showed a decline in episodic memory (recall with clues), while those who did not convert to dementia showed improvement in the same cognitive domain, according to the Wilcoxon test (Table 1). Memory decline is a characteristic of the early stages of dementia. Similar results were also reported by Albert et al.,^7^ who examined, in a longitudinal study, neuropsychological changes of 197 subjects with MCI and healthy participants at baseline. After four years, the participants who converted to dementia had greater decline in episodic memory when compared to the control group and to the elderly groups that were stable or showed cognitive decline over time.

The data were also treated qualitatively. Based on 1 SD declines presented by the sample on the neuropsychological tests, the subjects were divided into the following groups: No Decline, Memory Decline, Executive Functions Decline, and Multiple Functions Decline. Most of the elderly who converted to dementia were part of the Multiple Functions Decline group (Table 3). This group showed significant impairment in functioning. Most of the participants who converted to dementia presented baseline deficits in episodic memory and other cognitive functions, including global cognitive functioning, working memory storage, executive functions, language, and attention. That is, the elderly who converted to dementia had a similar baseline cognitive profile to older adults with amnestic and multiple domains MCI. The amnestic and multiple domains MCI profiles exhibit episodic memory impairment and changes in other cognitive functions while preserving the activities of daily living, and having no dementia.^2^ These profiles have a greater risk of converting to Alzheimer's disease or vascular dementia.^2^

By comparing the decline groups using the Mann-Whitney test, the Executive Functions Decline group stood out with significantly higher scores of depression symptoms after two years (Table 3) than both the Memory Decline and Multiple Functions Decline Groups. The relationship between depression and cognitive functions is thoroughly described in the literature.^21^^-^ 24 Overlapping symptoms often make it difficult to distinguish major depression from MCI or dementia.^25^ Depression is associated with cognitive deficits in aging and is considered a risk factor for Alzheimer's disease and vascular dementia.^21^^,^ 22 Cognitive dysfunction in information processing/attention, memory and executive function areas has been reported in patients with depression, and their neuropsychological patterns closely resemble those found in MCI, suggesting comorbidity between MCI and depression. In all age groups, depression is often accompanied by cognitive impairment, with deficits in the aspects cited above, with the most typical symptoms. Valid discrimination of the MCI cognitive profiles of subjects and patients with depression seems difficult to achieve without systematic follow-up assessments or considering other measures.^26^ Wong et al.^21^ examined the cognitive performance of 52 depressed patients. At baseline, 53.8% showed cognitive impairment on a screening tool for dementia (MoCA). After 6 months of treatment with medical experts, 61.5% showed improvement in depression symptoms and persistent cognitive impairment in 44.2% of the sample. In a study by De-Paula et al.,^27^ 274 healthy elderly adults with MCI and dementia were evaluated to investigate the association between depression and cognitive and functional performance in each group. In the group of non-demented older adults, depressed participants performed worse than non-depressed participants on the cognitive tests, with a greater effect being observed in executive function tests. There was a decrease in the association between cognition and depression in the MCI and dementia groups. An association between depression and executive functions was also observed in this study, confirming other reports in the literature.^21^^-^ 24

Most longitudinal studies of elderly adults with MCI have focused on the neuropsychological predictors of dementia and the rate of MCI conversion to dementia. Few recent studies have sought to identify the trajectory of cognitive decline in older adults with MCI, using different methodologies.^6^^,^ 7 The data showed different cognitive decline profiles in elderly adults with MCI and differences between them regarding depressive symptoms and rate of conversion to dementia. This study reinforces that, depending on the neuropsychological profile at baseline, conversion to dementia or other cognitive/behavior decline trajectory can be predicted. These data do not change the MCI definition, but provide a better understanding of the heterogeneity of MCI trajectory evolution.
